# Long-term oral prednisolone exposure in primary care for bullous pemphigoid: population-based study

**DOI:** 10.3399/BJGP.2020.0870

**Published:** 2021-10-05

**Authors:** Monica SM Persson, Karen E Harman, Kim S Thomas, Joanne R Chalmers, Yana Vinogradova, Sinead M Langan, Julia Hippisley-Cox, Sonia Gran

**Affiliations:** Centre of Evidence Based Dermatology, School of Medicine, University of Nottingham, Nottingham.; Centre of Evidence Based Dermatology, School of Medicine, University of Nottingham, Nottingham.; Centre of Evidence Based Dermatology, School of Medicine, University of Nottingham, Nottingham.; Centre of Evidence Based Dermatology, School of Medicine, University of Nottingham, Nottingham.; Division of Primary Care, University of Nottingham, Nottingham.; Department of Non-communicable Disease Epidemiology, London School of Hygiene and Tropical Medicine, London.; Nuffield Department of Primary Care Health Sciences, University of Oxford, Oxford.; Centre of Evidence Based Dermatology, School of Medicine, University of Nottingham, Nottingham.

**Keywords:** bullous pemphigoid, Clinical Practice Research Datalink, corticosteroid, prednisolone, prescriptions, primary health care

## Abstract

**Background:**

Oral prednisolone is the mainstay treatment for bullous pemphigoid, an autoimmune blistering skin disorder affecting older people. Treatment with moderate-to-high doses is often initiated in secondary care, but then continued in primary care.

**Aim:**

To describe long-term oral prednisolone prescribing in UK primary care for adults with bullous pemphigoid from 1998 to 2017.

**Design and setting:**

A prospective cohort study using routinely collected data from the Clinical Practice Research Datalink, a primary care database containing the healthcare records for over 17 million people in the UK.

**Method:**

Oral prednisolone exposure was characterised in terms of the proportion of individuals with incident bullous pemphigoid prescribed oral prednisolone following their diagnosis, and the duration and dose of prednisolone.

**Results:**

In total, 2312 (69.6%) of 3322 people with bullous pemphigoid were prescribed oral prednisolone in primary care. The median duration of exposure was 10.6 months (interquartile range [IQR] 3.4–24.0). Of prednisolone users, 71.5% were continuously exposed for >3 months, 39.7% for >1 year, 14.7% for >3 years, 5.0% for >5 years, and 1.7% for >10 years. The median cumulative dose was 2974 mg (IQR 1059–6456). Maximum daily doses were ≥10 mg/day in 74.4% of prednisolone users, ≥20 mg/day in 40.7%, ≥30 mg/day in 18.2%, ≥40 mg/day in 6.6%, ≥50 mg/day in 3.8%, and ≥60 mg/day in 1.9%.

**Conclusion:**

A high proportion of people with incident bullous pemphigoid are treated with oral prednisolone in UK primary care. Action is required by primary and second care services to encourage use of steroid-sparing alternatives and, where switching is not possible, ensure prophylactic treatments and proactive monitoring of potential side effects are in place.

## INTRODUCTION

Bullous pemphigoid is an autoimmune skin disease, characterised by the formation of intensely itchy blisters, which largely affects older people. Approximately 3500 people are diagnosed with bullous pemphigoid for the first time in England every year, and the diagnosis is associated with approximately three-times increased risk of death in the first 2 years.^1^^,^^2^ Additionally, the diagnosis is associated with increased risk of autoimmune conditions (for example, systemic lupus erythematosus), neurological conditions (for example, Parkinson’s disease), cardiovascular conditions (for example, hypertension), and other skin conditions (for example, psoriasis).^3^^–^^5^

Oral prednisolone has traditionally been the first-line systemic treatment for bullous pemphigoid for decades.^6^^,^^7^ In recent years, the benefit of safer alternatives has been demonstrated including super-potent topical corticosteroids and anti-inflammatory antibiotics (for example, doxycycline)^8^^,^^9^ but systemic steroids are still widely used. Although effective, prednisolone exposes an already vulnerable group to an increased risk of conditions such as osteoporosis and diabetes.^10^^,^^11^ Patients may be started on moderate-to-high doses of oral prednisolone in primary or secondary care settings. Patients referred onwards to secondary care for diagnosis and treatment of bullous pemphigoid typically have their long-term management shared jointly between primary and secondary care teams.

Characterising oral prednisolone exposure allows us to better understand the iatrogenic risks for people with bullous pemphigoid. The long-term use in this population is poorly understood, and the little available evidence is based on small studies involving hospital-based patients (Table 1).^12^^–^^22^ Routinely collected health data from primary care, in the form of the Clinical Practice Research Datalink (CPRD), provides an opportunity to address this research gap using a large population-based sample that is broadly representative of the UK.^23^ This study examines prescriptions for oral prednisolone issued in UK primary care for incident cases of bullous pemphigoid.

**Table 1.** Proportion of patients with bullous pemphigoid exposed to oral prednisolone as presented in published studies, presented alongside dose and duration information

**Publication**	**Country**	**Setting**	**Years**	***n***	**Treatment timing**	**Patients on oral prednisolone, %**	**Additional dose and duration detail**
Balestri *et al* (2018)^12^	Italy	Clinic	2008–2012	53	Initial	96.2	Dose range: 0.5–0.75 mg/kg/day
Kremer *et al* (2017)^13^	Israel	Hospital	2008–2014	104	Initial	78.1	Mean dose: 57.3 mg/day (range 30–70 mg/day)
Zhang *et al* (2013)^14^	China	Hospital	2005–2010	94	Initial	85.0	Maximum dose: range 20–80 mg/day
Esmaili *et al* (2012)^15^	Iran	Hospital	1987–2007	122	Initial	73.8	Mean dose: 60.38 mg/day (SD 21.21, range 5–120 mg/day)
Kulthanan *et al* (2011)^16^	Thailand	Clinic	1991–2009	58	Initial	89.7	Mean cumulative dose to achieve remission: 0.05 g/kg
Serwin *et al* (2007)^17^	Poland	Hospital, clinic	2000–2005	35	Initial	68.6	Dose range: 40–60 mg/day
Nanda *et al* (2006)^18^	Kuwait	Clinic	1991–2005	41	Initial	100	—
Tan and Tay (2018)^19^	Singapore	Hospital	2004–2012	100	Any time	96.0	Mean duration: 11.6 months (range 1 week to 60 months)
Wong and Chua (2002)^20^	Singapore	Hospital	1998–1999	59	Initial	76.0	Mean dose: 31.2 mg/day (range 15–60 mg/day) when used as monotherapy
Chang *et al* (1996)^21^	Taiwan	Hospital	1977–1994	86	Initial	83.7	Mean dose: 54.1 mg/day
Garcia-Doval *et al* (2005)^22^	Spain	Hospital	1998–2003	26	Unclear	53.9	Mean daily dose at start of therapy: 34 mg (SD 9.8, range 20–50 mg/day) Mean duration: 20 months (SD 12)

*SD = standard deviation.*

## METHOD

### Study design and data source

This was a prospective cohort study using routinely collected health data from the CPRD. The CPRD is a longitudinal database of UK general practices containing the anonymised diagnosis, referral, prescription, and vaccination data of approximately 17 million people, with a current coverage of approximately 2.7 million (4%) of the UK population. Although bullous pemphigoid is predominantly diagnosed in secondary care, the diagnosis is subsequently transcribed from discharge or specialist clinic letters into the CPRD using Read codes.^24^

**Table:** How this fits in

Bullous pemphigoid is an autoimmune blistering skin disorder that generally affects older people and is associated with a threefold increase in mortality. Although oral prednisolone has been considered the mainstay of treatment for decades, its long-term use in primary care is poorly characterised. This study found that 70% of people with incident bullous pemphigoid were prescribed oral prednisolone in primary care, at considerable doses and durations of exposure. As they may be on oral prednisolone for prolonged periods of time, conversations between primary and secondary care physicians involved in their care should address steroid-sparing alternatives and, when switching is not possible, ensure prophylactic treatment (for example, bone-sparing treatments) and proactive monitoring of side effects are in place.

For this study only practices recording data using VISION software (CPRD GOLD) were used. The data in the CPRD have repeatedly been shown to be of good research quality.^25^ At the practice level, participating practices are audited to confirm data quality. At the patient level, records are assessed and data checks are conducted to ensure that the record meets prespecified quality standards.

This work follows the REporting of studies Conducted using Observational Routinely collected health Data (RECORD) guidelines.^26^ The data can be requested from www.cprd.com.

### Study population

The study population comprised adult males and females with incident bullous pemphigoid diagnoses between January 1998 and December 2017, selected using previously described methods.^1^^,^^27^ In short, a validated algorithm was implemented to identify people with a code for bullous pemphigoid (M145), pemphigoid (M145.00), or pemphigoid NOS (not otherwise specified, M145z00) in their clinical records. This approach has a positive predictive value of 93.2% (95% confidence interval [CI] = 91.3% to 94.8%).^27^ The bullous pemphigoid index date was the date the diagnosis was first recorded.

To identify people for whom prednisolone might have been prescribed for alternative indications, only people with at least 12 months’ data before the bullous pemphigoid index data were eligible. In order to allow sufficient time to capture prescriptions (under the assumption that initial treatment would be prescribed in secondary care and therefore would not appear in the CPRD), people with <6 months’ follow-up after their bullous pemphigoid index date were excluded.

### Observation period

People were followed up from their bullous pemphigoid index date until the earliest of the date: 1) the person left the practice; 2) the person died; 3) the practice last contributed data to the CPRD; or 4) 31 December 2017.

### Oral prednisolone prescriptions

All prescriptions for oral prednisolone (see Supplementary Table S1 for Read codes) during the observation period were identified. This study focused only on prescriptions issued after the bullous pemphigoid index date based on the assumption that these would reflect long-term management following the diagnosis.

Prednisolone dose and duration were extracted when available and imputed when missing. These data were often missing when prescriptions were issued with information restricted to the free-text field, such as ‘Take as indicated by your dermatologist’. Implausible and missing values were handled using the DrugPrep algorithm,^28^ with the decisions described and validated by Joseph *et al*^29^ (see Supplementary Table S2). Cleaning oral glucocorticoid prescriptions in this way has been found to have a sensitivity of 84.2% (95% CI = 68.7% to 94.0%) and a specificity of 87.5% (95% CI = 73.2% to 95.8%) for predicting patient-reported current glucocorticoid use.^29^

### Alternate indications for oral prednisolone

Oral prednisolone can be prescribed for other indications besides bullous pemphigoid, such as rheumatoid arthritis or asthma. To understand the proportion of people who may have been prescribed prednisolone for reasons other than bullous pemphigoid, those with a Read code for an alternative indication in the 12 months preceding their bullous pemphigoid index date were identified. The code lists were drawn from Kuan *et al*^30^ (see Supplementary Table S3).

### Statistical analysis

The proportion of people prescribed oral prednisolone following their bullous pemphigoid index date was determined. The proportion of prednisolone users who may have been prescribed prednisolone for an alternative indication was determined (that is, people with a Read code for an alternate indication and a prescription for oral prednisolone in the 12 months preceding bullous pemphigoid).

For each prednisolone user, the number of prescriptions, total follow-up time, followup time on prednisolone, and proportion of follow-up on prednisolone were determined. The duration of continuous exposure, defined as prescriptions with <15 days between the end of one and the start of the next, was determined for each patient. The number of periods of continuous exposure were determined for each patient and summarised across the population. The proportion continuously exposed for longer than 3 months, 1 year, 3 years, 5 years, and 10 years were determined. For these, the denominator included only people with follow-up longer than the duration of interest (that is, longer than 3 months, 1 year, 3 years, 5 years, and 10 years, respectively).

Finally, the doses of oral prednisolone were examined. The median daily dose for all prescriptions was determined. The proportion of prednisolone users prescribed ≥10 mg/day, ≥20 mg/day, ≥30 mg/day, ≥40 mg/day, ≥50 mg/day, and ≥60 mg/day was determined. The cumulative dose of prednisolone throughout the whole observation period was calculated for each patient, and the median determined across the population. The average dose while on prednisolone was determined by dividing the cumulative dose by the duration of exposure.

A sample size calculation was not conducted as this was a descriptive study using all available data. Population summary measures were presented as the median (interquartile range [IQR]) of continuous variables or number (proportion) for each categorical measure. Analyses were conducted with Stata 16.

## RESULTS

### Study population

**Figure 1.**
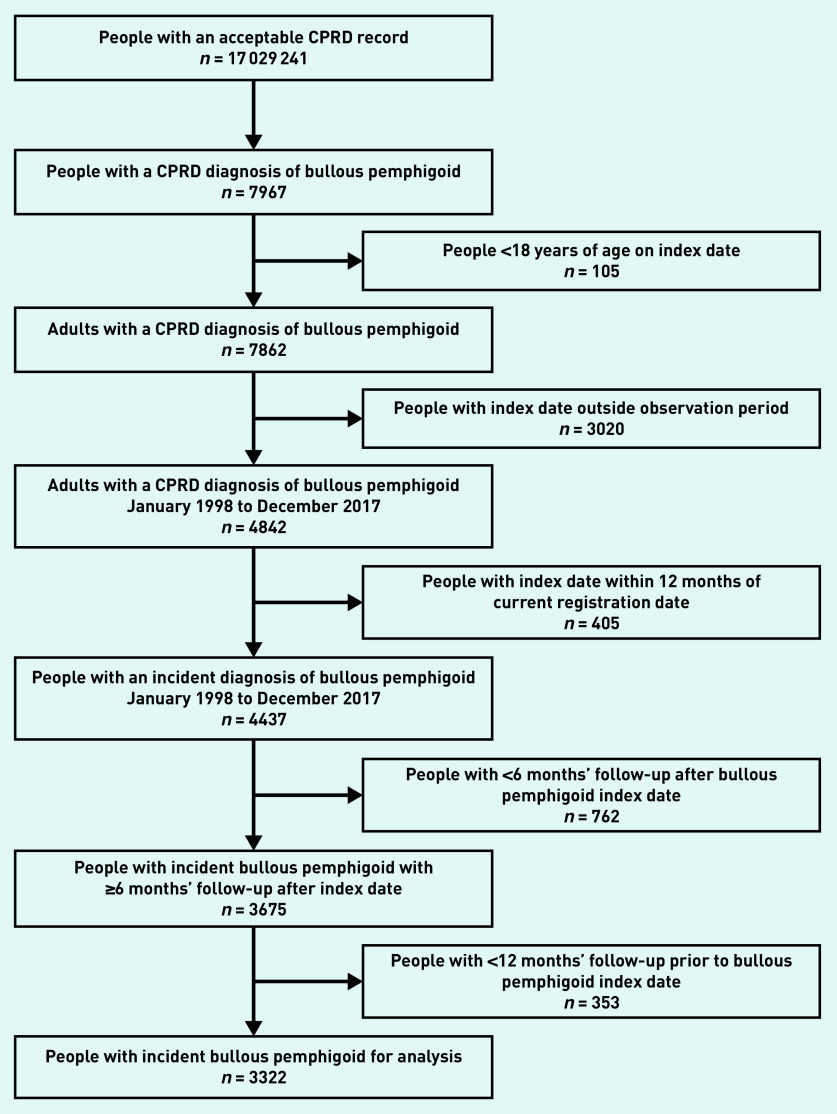
*Identification of the study population of adults with incident bullous pemphigoid from the CPRD. CPRD = Clinical Practice Research Datalink.*

There were 4437 people with incident bullous pemphigoid in the study period (Figure 1). The 762 people with <6 months’ follow-up after their index date were excluded from the study, of whom 499 died. A further 353 people without 12 months of data before their index date were also excluded. The study population therefore comprised 3322 people with incident bullous pemphigoid with at least 12 months of data before and 6 months of data after their index date. They were identified from 667 practices, with a median of four people with bullous pemphigoid for each GP practice.

The median age at first recording of bullous pemphigoid was 79.7 years (IQR 71.6–86.0) and 1858 (55.9%) were female. Median duration of follow-up was 3.1 years (IQR 1.5–5.7) and ranged from 6 months to 18 years. Overall, the population represents 13 758.2 person–years of follow-up (data not shown).

### Prednisolone users

Overall, 2312 (69.6%) people were prescribed oral prednisolone after their bullous pemphigoid index date. They were followed for 9506.0 person–years, of which 9.3 person–years were on prednisolone. The median number of months individuals spent on prednisolone was 10.6 (IQR 3.4–24.0), representing a median of 0.11% (IQR 0.02–0.24) of their follow-up. The median number of prescriptions for prednisolone for each patient was 15 (IQR 8–26) (data not shown).

Of the prednisolone users, only 321 (13.9%) had complete data for all prescriptions. For the remaining patients, the dose, start date, or treatment duration were imputed for at least one prescription. Eighty-eight (3.8%) of the prednisolone users had a Read code for an alternate indication (for example, rheumatoid arthritis) and a prednisolone prescription in the 12 months before bullous pemphigoid (data not shown).

### Duration of continuous exposure

**Figure 2.**
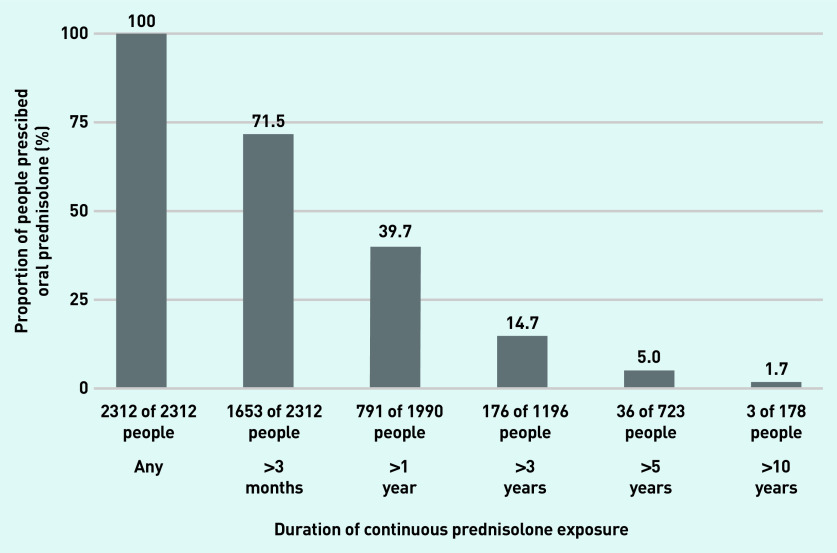
***Proportion of prednisolone users continuously exposed to oral prednisolone for longer than 3 months, 1 year, 3 years, 5 years, and 10 years.****^a^* *^a^****The denominator for duration includes only people with follow-up durations exceeding the duration of interest.***

The median number of periods of continuous exposure for a patient was 2 (IQR 1–3) and periods ranged from 1 day to 12 years duration. Overall, 71.5% of prednisolone users were on prednisolone continuously for >3 months, 39.7% for >1 year, 14.7% for >3 years, 5.0% for >5 years, and 1.7% for >10 years (Figure 2).

### Oral prednisolone doses

**Figure 3.**
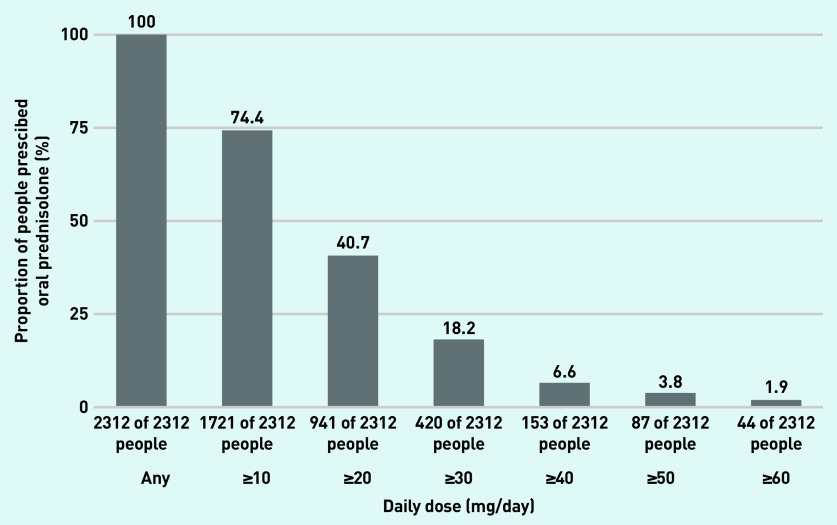
***Proportion of prednisolone users prescribed a maximum daily dose of oral prednisolone*** ≥***10 mg,*** ≥***20 mg,*** ≥***30 mg,*** ≥***40 mg,*** ≥***50 mg,*** ≥***60 mg at any point during the observation period.***

The median daily dose across all prescriptions was 10 mg/day (IQR 5–13). There were 1721 (74.4%) prednisolone users who were prescribed a maximum dose of ≥10 mg/day, 941 (40.7%) were prescribed ≥20 mg/day, 420 (18.2%) ≥30 mg/day, 153 (6.6%) ≥40 mg/day, 87 (3.8%) ≥50 mg/day, and 44 (1.9%) ≥60 mg/day (Figure 3) at any point during the observation period.

The median cumulative dose during followup was 2974 mg (IQR 1059–6456). Focusing only on the duration of follow-up where the person was on a prednisolone prescription, the average daily dose was <2.5 mg/day for 11 (0.5%) people, 2.5–5.0 mg/day for 152 (6.6%) people, 5.0–7.5 mg/day for 351 (15.2%) people, and >7.5 mg/day for 1798 (77.8%) people (data not shown).

## DISCUSSION

### Summary

This study explored oral prednisolone prescribing for people with bullous pemphigoid in UK primary care. Of all people with new diagnoses of bullous pemphigoid identified from 1998 to 2017, 69.6% were prescribed oral prednisolone in primary care following their diagnosis. Under the assumption that the initial high-dose regimens of prednisolone are more likely to be issued in dermatology clinics rather than in primary care, and ongoing prescriptions may not exclusively be issued in primary care, these findings likely underestimate both the duration of exposure and dosages of prednisolone, and should be viewed as the minimum exposure.

This study found that 71.5% of prednisolone users were exposed to prednisolone continuously for >3 months, and 39.7% were on prednisolone for >1 year. A small subset (1.7%) were on prednisolone continuously for >10 years. Most patients (74.4%) received daily doses ≥10 mg/day at some point. The prescriptions totalled a median cumulative dose of 2974 mg throughout the study period and, for 77.8% of the patients, an average daily dose of >7.5 mg/day during active periods of prescriptions.

Despite this study only presenting the minimum estimated exposure, these levels are sufficient to place people with bullous pemphigoid at risk of corticosteroid-associated adverse events.^31^^,^^32^ Strict monitoring and proactive management are required to minimise the risks to this population.

### Strengths and limitations

Strengths of the present work include a large sample size, people with bullous pemphigoid identified from a source population that is broadly representative of the UK,^23^ and the implementation of validated methods for identifying bullous pemphigoid and preparing the prescription data. The algorithm used to identify people with bullous pemphigoid has a positive predictive value of 93.2%,^27^ thus indicating that the population analysed likely have bullous pemphigoid. The approach to preparing the prescriptions is validated and shown to accurately classify current oral corticosteroid status (on/off) for 86% of patients.^29^ Daily dose estimates generated from this approach are imprecise but not significantly biased, with a mean absolute difference in estimated and reported doses of 3.2 (standard deviation [SD] 4.2) mg/day.^29^ Finally, the authors of the current study believe it largely comprises individuals who were prescribed prednisolone for bullous pemphigoid rather than for other indications. Only 3.8% of the prednisolone users had: 1) a record for an alternative indication for oral prednisolone; and 2) a prescription for oral prednisolone in the 12 months preceding bullous pemphigoid.

Limitations largely relate to the nature of the data used. First, although validated approaches for identifying patients and exposure were used, the findings may be subject to misclassification and measurement error. This is further compounded by the large proportion of missing data, affecting at least one prescription for 86.1% of people. Second, only prescriptions issued in primary care could be examined. As a result, it was only possible to present the minimum estimated exposure to prednisolone. In addition, the absence of information regarding the timing and duration of secondary care follow-up meant that this study could not describe the interplay between primary and secondary care prescribing of prednisolone. Third, it was not possible to capture and describe tapering regimens for prednisolone because of insufficient granularity in the data. Fourth, only oral prednisolone was examined and other systemic oral corticosteroids were not considered, such as betamethasone, and the study may therefore have underestimated total corticosteroid exposure. Finally, exclusion of people with <6 months’ follow-up may have limited the external validity of the sample as death was the commonest reason for insufficient follow-up. However, it was felt that including people with <6 months’ follow-up would artificially lower the estimated exposure to prednisolone as there was insufficient time for the prescriptions to pass from dermatology clinics to general practice.

### Comparison with existing literature

To the authors’ knowledge, this is the first population-based cohort study to examine the prescribing patterns of oral prednisolone for bullous pemphigoid in UK primary care. Recent evidence on oral corticosteroid use for bullous pemphigoid in the UK is based on a national audit completed by members of the British Association of Dermatologists. In this, 85.5% (*n* = 448/524) of those with bullous pemphigoid diagnoses were prescribed an oral corticosteroid by their dermatologist at some point during their management.^33^ The current study found that a more modest 69.6% of people are prescribed prednisolone in primary care, although this discrepancy may be largely attributed to the different settings. The higher proportion from the British Association of Dermatologists audit suggests that some people are exposed to prednisolone only in secondary, and not primary, care. It may be that these people commence oral prednisolone, which is then stopped in favour of an alternative treatment, because of disease resolution, or death. Alternatively, they may continue to be prescribed oral corticosteroids in secondary care.

Worldwide, several studies have reported oral prednisolone use in people with bullous pemphigoid. The current findings are largely in keeping with the proportion of prednisolone users in the previous literature, although with substantially lower doses than elsewhere (Table 1). Again, this may be because of differences in the setting and timing of prescriptions (that is, initial versus long-term treatment). In clinical practice, oral corticosteroids are used with a slowly reducing regimen for many months. Previous studies have captured the high initial doses prescribed. This work has extended beyond this initial period and captured high maximum doses (≥60 mg/day in some) potentially indicative of initial doses and lower maintenance doses (for example, median daily doses <10 mg/day), potentially reflecting tapering regimens. Further interpretation of earlier evidence is hindered by the small population sizes and the largely hospital-based setting of previous research that generally did not extend beyond the initial management.

### Implications for research and practice

Those with bullous pemphigoid are a clinically vulnerable group because of their older age (median age: 79.7 years) and significant comorbidities, including hypertension, diabetes mellitus, heart failure, and osteoporosis.^34^ There is therefore a need for careful consideration of additional risks for this population. There is evidence of a dose-dependent relationship between the cumulative dose of prednisolone and sleeping problems, acne, skin bruising, mood problems, cataracts, hyperglycaemia, and bone fractures.^35^ Focusing specifically on fracture risk, a daily dose of 7.5 mg/day approximately doubles the chance of developing a hip and vertebral fracture compared with <2.5 mg/day.^32^ This risk is evident even within 3–6 months of starting.^31^ As such, clinicians are exposing people with bullous pemphigoid to substantial iatrogenic risks as a result of prednisolone prescribing. Although these risks are outweighed in the short term by the urgent need to control the disease, this work shows that the exposure to prednisolone extends beyond the initial regimens prescribed in dermatology clinics.

Although GPs will often not be involved in the initial aggressive management of bullous pemphigoid, they may be tasked with prednisolone prescribing in the longer term. The authors urge clinicians to be mindful that this population, who may already be frail because of their age and significant comorbidities, may be on large doses of prednisolone for substantial periods of time. Strict monitoring and careful consideration of prophylactic treatments, such as bone-protection therapies, are essential for their long-term management. In addition, conversations between primary and secondary care should take place to consider steroid-sparing alternative treatments such as doxycycline. In 2017, doxycycline was shown to be non-inferior to oral corticosteroids for the management of bullous pemphigoid.^8^ This will likely have an impact on clinicians’ practices, but such changes will not have been captured by the present work (observation period 1998–2017). Further research may be needed to re-explore systemic steroid prescribing in patients post-2017.

Future research should also expand on this current study to examine steroid-related outcomes (for example, hip and pelvis fractures) in people with bullous pemphigoid, and to determine whether adequate monitoring and prescription of prophylactic treatment (for example, bisphosphonates) occur.
